# Comparative cytogenetic analysis between *Lonchorhina aurita* and *Trachops cirrhosus* (Chiroptera, Phyllostomidae)

**DOI:** 10.1590/S1415-47572009005000095

**Published:** 2009-12-01

**Authors:** Helen Maria Duarte do Rêgo Barros, Cibele Gomes de Sotero-Caio, Neide Santos, Maria José de Souza

**Affiliations:** Laboratório de Genética e Citogenética Animal, Departamento de Genética, Centro de Ciências Biológicas, Universidade Federal de Pernambuco, Recife, PEBrazil

**Keywords:** bats, chromosome banding, fluorochromes, NOR

## Abstract

Phyllostomidae comprises the most diverse family of neotropical bats, its wide range of morphological features leading to uncertainty regarding phylogenetic relationships. Seeing that cytogenetics is one of the fields capable of providing support for currently adopted classifications through the use of several markers, a comparative analysis between two Phyllostomidae species was undertaken in the present study, with a view to supplying datasets for the further establishment of Phyllostomidae evolutionary relationships. Karyotypes of *Lonchorhina aurita* (2n = 32; FN = 60) and *Trachops cirrhosus* (2n = 30; FN = 56) were analyzed by G- and C-banding, silver nitrate staining (Ag-NOR) and base-specific fluorochromes. Chromosomal data obtained for both species are in agreement with those previously described, except for X chromosome morphology in *T. cirrhosus*, hence indicating chromosomal geographical variation in this species. A comparison of G-banding permitted the identification of homeologies in nearly all the chromosomes. Furthermore, C-banding and Ag-NOR patterns were comparable to what has already been observed in the family. In both species CMA_3_ /DA/DAPI staining revealed an R-banding-like pattern with CMA _3_ , whereas DAPI showed uniform staining in all the chromosomes. Fluorochrome staining patterns for pericentromeric constitutive heterochromatin (CH) regions, as well as for nucleolar organizing regions (NORs), indicated heterogeneity regarding these sequences among Phyllostomidae species.

## Introduction

The family Phyllostomidae, which comprises the New World leaf-nosed bats, is considered the third largest of the order Chiroptera. This family is the most diverse group among Neotropical bats, with approximately 56 genera and 141 species (Baker *et al.*, 2003; Simmons, 2005). Phyllostomidae bats exhibit wide variation in morphological features, and are adapted to a extensive range of ecological niches, with dietary specialization which includes fruit, nectar, pollen, insects, vertebrates and blood. This great diversity has been problematic for systematics, and concurs to hindering efforts to reconstruct the phylogenetic history of the family (Wetterer *et al.*, 2000; Jones, 2002; Baker *et al.*, 2003).

The subfamily Phyllostominae is one of the groups which has been questioned by researchers, but without consensus. Several authors agree that this subfamily is not a monophyletic group, although only recently has a new proposal been made as to its subdivision. Baker *et al.* (2003), on analyzing mtDNA sequence data, grouped this information together with previous phylogenies based on the *RAG2* gene (Baker *et al.*, 2000), morphology (Wetterer *et al.*, 2000) and karyotypes (Baker *et al.*, 1973, 1989; Baker and Bass, 1979), to suggest a classification with 56 genera in 11 subfamilies for the Phyllostomidae. In this classification, members were distributed among five subfamilies: Macrotinae, Micronycterinae, Lonchorhininae, Phyllostominae and Glyphonycterinae. Lonchorhininae, which is comprised of a single genus (*Lonchorhina*), diverged before the radiation of Phyllostominae and nectarivorous bats, appearing as a basal branch relative to Phyllostominae.

Cytogenetic studies constitute an important approach for understanding phylogenetic relationships among bats. By comparing banding patterns and the localization and constitution of different markers, it has been possible to characterize several taxa and develop hypotheses on evolutionary relationships, as well as models of chromosomal evolution (Baker *et al.*, 1989; Baker, 2006).

Thus, in this work, chromosomal features of *Lonchorhina aurita* (Lonchorhininae) and *Trachops cirrhosus* (Phyllostominae) were studied by conventional analysis, G- and C-banding, staining with silver nitrate and base-specific fluorochromes (CMA_3_ and DAPI) in order to establish mutual cytogenetic differences. These data will be helpful in understanding the chromosome structure and evolution of the family Phyllostomidae, as well as systematic aspects and phylogenetic relationships among members.

## Materials and Methods

Chromosome analyses were carried out on 12 specimens (seven males and five females) of *Lonchorhina aurita* (Tomes, 1863) and eight specimens (four males and four females) of *Trachops cirrhosus* (Spix, 1823). *L. aurita* individuals were captured in the locality of Toritama (8° 0' 24” S, 36° 3' 24” W) and *T.cirrhosus* specimens were captured in the Reserva Biológica de Saltinho, Rio Formoso (8° 39' 49” S, 35° 9' 31” W), both in the state of Pernambuco, northeastern Brazil. Metaphase chromosome preparations were obtained from bone-marrow cells according to conventional procedures.

Silver staining and G- and C-banding procedures were undertaken through routine cytogenetic techniques, according to Howell and Black (1980), Seabright (1971) and Sumner (1972), respectively. Triple staining CMA_3_/DA/DAPI was carried out according to Schweizer (1980) with various modifications (Santos and Souza, 1998a). For sequential staining (AgNO_3_/CMA_3_/DAPI), the slides stained with silver nitrate were distained after photographing (Dos Santos Guerra, 1991) and re-stained with CMA_3_/DA/DAPI.

Photomicrographs were taken by means of a Leica DMLB photomicroscope for conventional, silver staining and fluorescence staining. G- and C-banding were captured by a CytoVision image capture system.

## Results

The karyotype of *L. aurita* presented the diploid number 2n = 32,XX;XY and the fundamental number FN = 60, and included metacentric (1, 4, 6, 8, 9, 11, 13 and 15), submetacentric (2, 3, 5 and 7) and subtelocentric (10, 12 and 14) chromosomes. The X chromosome was medium-sized submetacentric and the Y minute. In the *T. cirrhosus* karyotype, the diploid number was 2n = 30, XX;XY and FN = 56, and was comprised of metacentric (1, 6, 8, 10, 13 and 14), submetacentric (2, 3, 4 and 5) and subtelocentric (7, 9, 11 and 12) chromosomes. The X and Y chromosomes were acrocentric.

**Figure 1:**
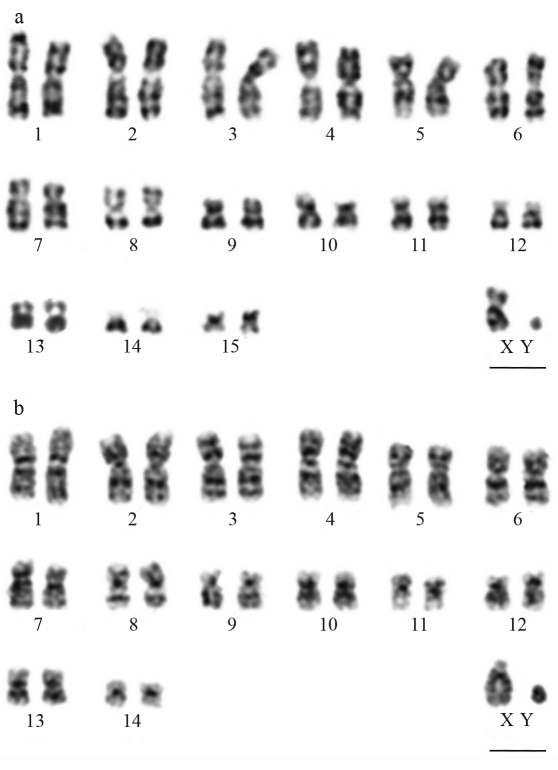
GTG-banding pattern. (**a**) *Lonchorhinaaurita*, (**b**) *Trachops cirrhosus*. Bar = 5 μm.

**Figure 2:**
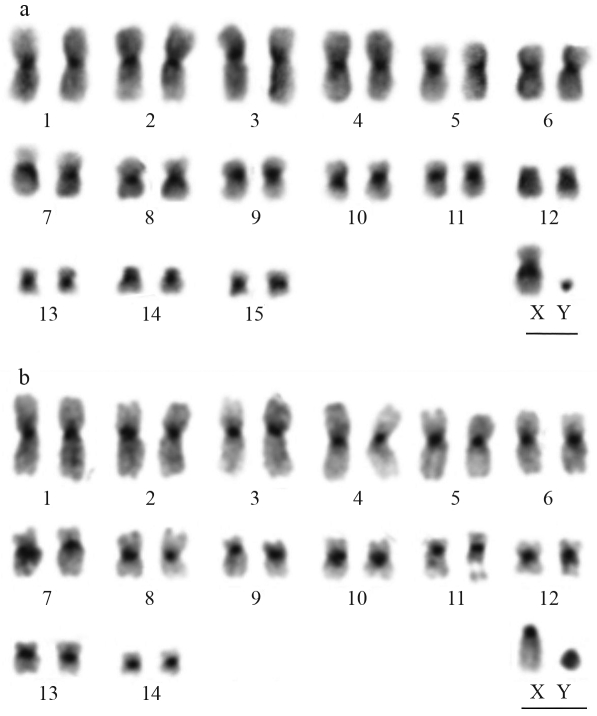
CBG-banding pattern. (**a**) *Lonchorhinaaurita*, (**b**) *Trachops cirrhosus*. Bar = 5 μm.

The G-banding pattern disclosed the precise identification of all chromosome pairs. Comparative banding analysis inferred homeologies between the two species in pairs 1 to 3 and 5 to 8 (Figures 1a and 1b). Furthermore, in *L. aurita* the chromosome pairs 9, 10, 11 and 15 appeared to correspond to pairs 13, 12, 10 and 14 in *T. cirrhosus*, respectively. C-banding revealed constitutive heterochromatin (CH) in the pericentromeric regions of all the autosomes and the X chromosome, whereas the Y chromosome was almost completely heterochromatic in both species (Figures 2a and 2b).

**Figure 3:**
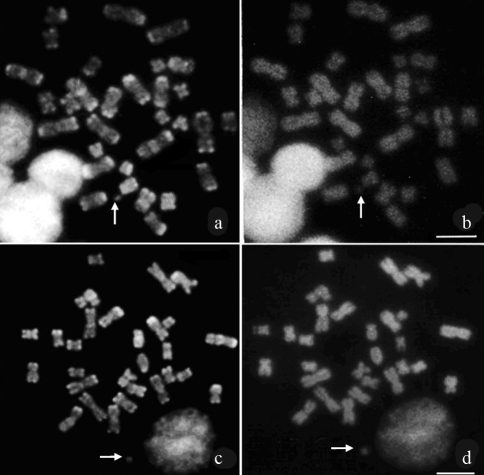
Metaphases after CMA_3_/DA/DAPI staining. (**a-b**) *Lonchorhina* aurita, (**c-d**) *Trachops cirrhosus*: CMA_3_ (**a**, **c**) and DAPI (**b**, **d**). Arrows indicate the Y chromosome. Bar = 5 μm.

Triple staining CMA_3_/DA/DAPI in these species revealed an R-banding-like pattern with the CMA_3_ dye (GC-rich regions) (Figures 3a and 3c), and uniform staining of all chromosomes with DAPI (Figures 3b and 3d). In addition, CMA_3_-positive blocks were observed in the pericentromeric region of some chromosomes, thereby indicating the GC-richness of CH.

**Figure 4:**
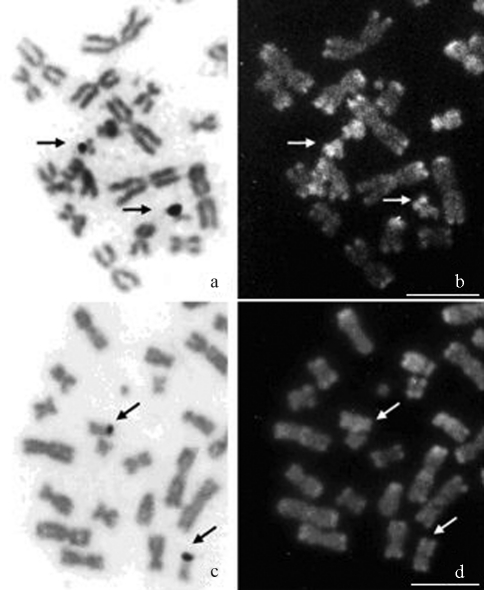
Partial metaphases after AgNO_3_/CMA_3_/DAPI sequential staining. (**a-b**) *Lonchorhinaaurita*, (**c-d**) *Trachops cirrhosus*. AgNO_3_ staining (**a**, **c**), arrows pointing to NOR sites. Note that NORs are CMA_3_ positive in *L.aurita* (**b**) and CMA_3_ neutral in *T. cirrhosus* (**d**). Bar = 5 μm.

Staining with silver nitrate (AgNO_3_) revealed a single pair of NORs located at the secondary constriction in both species: in the short arm of pair 13 in *L. aurita* (Figure 4a) and in the long arm of pair 11 in *T. cirrhosus* (Figure 4c). The signals resulting from sequential staining AgNO_3_/CMA_3_/DAPI, revealed CMA_3_ positive NORs in *L. aurita* (Fig. 4b), whereas these regions were CMA_3_ neutral in *T. cirrhosus* (Figure 4d).

## Discussion

Our data regarding diploid number, chromosome morphology and sex determination system obtained for both *L. aurita* and *T.cirrhosus* are in agreement with those previously described, except for the X chromosome in *T. cirrhosus*. We observed an acrocentric X in specimens from Pernambuco, Brazil, although this has been described as subtelocentric in individuals from Mexico and Trinidad (Baker, 1967; Hsu *et al.* 1968; Baker and Hsu, 1970).

The majority of Phyllostomidae species have a biarmed X chromosome (metacentric, submetacentric or subtelocentric) this condition being considered basal for the family (Rodrigues *et al.*, 2003). Acrocentric morphology of the X chromosome has been described in only three other Phyllostomidae species, *Micronycteris hirsuta* (Micronycterinae), *Mesophylla macconnelli* and *Vampyressa pusilla* (Stenodermatinae) (Baker and Hsu, 1970; Baker *et al.*, 1973; Gardner, 1977). However, despite having encountered the same morphology, we suggest that the acrocentric morphology of the X chromosome in *T. cirrhosus* (Phyllostominae) is an apomorphic character that has evolved independent of the condition observed in the three aforementioned species, as they are distantly related. The most probable event involved in the morphological change of the X chromosome in *T. cirrhosus* could be pericentric inversion occurring in an ancestral metacentric or submetacentric X.

The CH in Phyllostomidae is generally located in the pericentromeric regions of chromosomes (Varella-Garcia *et al.*, 1989), as observed in *L. aurita* and *T. cirrhosus*. However, additional CH blocks have been found in interstitial and telomeric regions in several species, notably *Carollia perspicillata*, *Choeroniscus minor*, *Glossophaga soricina*, *Artibeus lituratus*, *A. planirostris*, *A. jamaicencis*, *A. cinereus*, *Sturnira lilium*, *Platyrrhinus lineatus*, *Uroderma magnirostrum*, *U. bilobatum*, *Diaemus youngi*, *Desmodus rotundus* and *Diphylla ecaudata* (Varella-Garcia *et al.*, 1989; Souza and Araújo, 1990; Santos and Souza, 1998a, 1998b; Neves *et al.*, 2001; Santos *et al.*, 2001; Silva *et al.*, 2005). The Y chromosomes of *L. aurita* and *T. cirrhosus* were almost entirely heterochromatic, which is a common pattern in Phyllostomidae species (Varella-Garcia *et al.*, 1989; Souza and Araújo, 1990).

The occurrence of one pair of NORs located in secondary constrictions of chromosomes in *L. aurita* and *T. cirrhosus* seems to be an ancestral condition among phyllostomid bats (Morielle and Varella-Garcia, 1988; Santos *et al.*, 2002). NOR staining by GC-specific fluorochromes, as observed in *L. aurita*, has also been discerned in *Artibeus lituratus*, *A. jamaicencis*, *Desmodus rotundus* and *Diphylla ecaudata*, although these regions were CMA_3_ neutral in *Carollia perspicillata*, *Phyllostomus discolor* and *T. cirrhosus*. This indicates heterogeneity regarding the base composition of intergenic regions related to NORs among species of the family Phyllostomidae (Santos and Souza, 1998a, 1998b; Santos *et al.*, 2001.

In *L. aurita* and *T. cirrhosus* karyotypes, CMA_3_ staining resulted in a pattern similar to R-banding, although, a G-band-like pattern was not observed with DAPI staining. In both species, the pericentromeric CH regions of some chromosomes presented positive staining with CMA_3_ (CMA_3_^+^). In certain species, such as *Carollia perspicillata*, the presence of euchromatic bands (R- and G-bands) and heterochromatin heterogeneity (CMA_3_-positive, DAPI-positive and CMA_3_/DAPI- neutral) after CMA_3_/DA/DAPI staining has been observed (Santos and Souza, 1998a). On the other hand, the CH in three species of *Artibeus* (*A. lituratus*, *A. jamaicencis* and *A. cinereus*), as well as *Desmodus rotundus* and *Diphylla ecaudata*, indicated no AT- or CG- richness after staining with these dyes (Santos and Souza, 1998b; Santos *et al.*, 2001). Such a differential response to GC- and AT-specific fluorochromes in several Phyllostomidae species is a result of variability in heterochromatin composition within the family (Santos *et al.*, 2001).

In the family Phyllostomidae, it is common to use the karyotype of *Macrotus waterhousii* as a reference for the numbering system, since it is believed to represent the ancestral karyotype for the family (Baker, 2006). From the present work, it is obvious that the two species analyzed share considerable homeologies, with 11 identical chromosome pairs. Their karyotypes are highly derived when compared to the ancestral state (see Baker, 1979 for *M. waterhousii* standard reference G-banded karyotype). However, several chromosomal arms in *M. waterhousii* can be recognized as being homeologous to arms in the karyotypes of the two studied species. This gives support to the inference that the evolutionary trend in Phyllostomidae appears to lead to a reduction in diploid number by centric fusion events, with retention of the linkage groups. Furthermore, these 11 chromosomes were probably present in the ancestor before the radiation of *Lonchorhina*, the common ancestor of Phyllostominae (*sensu* Baker *et al.* 2003) and nectarivorous bats.

There are three biarmed elements, recognizable in the *M. waterhousii* karyotype, which remained unchanged in *L. aurita* and *T. cirrhosus*. These chromosomes correspond to *M. waterhousii* arms 6/7, 25/26, and 15/16 (Pairs 8, 9 and 11 of *L. aurita* and 8, 13 and 10 of *T. cirrhosus*). They have been described as unchanged in members of different subfamilies (*i.e.* Desmodontinae, Baker *et al.* 1988; Glossophaginae, Baker and Bass, 1979; and other Phyllostominae species, Patton and Baker, 1979), thereby indicating that they were already present in the common ancestor of all phyllostomid bats.

We have been unable to detect homeologies among some chromosomes for the studied species, and the banding pattern of distinct arms of these chromosomes is such as not to allow us to be certain of their correspondence. It is likely that most of these arms have undergone inversion prior to translocation, thereby hindering the identification of the rearrangements involved in karyotypic changes between these bats.

Members of the Phyllostomidae family have conserved karyotypes but show intergeneric variability, making a comparative analysis using classical banding difficult (Baker, 1979; Pieczarka *et al.*, 2005). However, further comparative chromosomal studies with molecular cytogenetic techniques based on fluorescence *in situ* hybridization (FISH) are expected to provide a better understanding of the karyotypic changes that have occurred during the evolution of this family, as well as the phylogenetic relationships among the members of this complex group of bats.
